# Evolution-Guided Structural and Functional Analyses of the HERC Family Reveal an Ancient Marine Origin and Determinants of Antiviral Activity

**DOI:** 10.1128/JVI.00528-18

**Published:** 2018-06-13

**Authors:** Ermela Paparisto, Matthew W. Woods, Macon D. Coleman, Seyed A. Moghadasi, Divjyot S. Kochar, Sean K. Tom, Hinissan P. Kohio, Richard M. Gibson, Taryn J. Rohringer, Nina R. Hunt, Eric J. Di Gravio, Jonathan Y. Zhang, Meijuan Tian, Yong Gao, Eric J. Arts, Stephen D. Barr

**Affiliations:** a Western University, Schulich School of Medicine and Dentistry, Department of Microbiology and Immunology, London, Ontario, Canada; Ulm University Medical Center

**Keywords:** HERC3, HERC4, HERC5, HERC6, restriction factor, HIV-1, positive selection, antiviral, retroviruses, interferon, HERC, evolution, human immunodeficiency virus, innate immunity, interferons, intrinsic immunity, simian immunodeficiency virus

## Abstract

In humans, homologous to the E6-AP carboxyl terminus (HECT) and regulator of chromosome condensation 1 (RCC1)-like domain-containing protein 5 (HERC5) is an interferon-induced protein that inhibits replication of evolutionarily diverse viruses, including human immunodeficiency virus type 1 (HIV-1). To better understand the origin, evolution, and function of HERC5, we performed phylogenetic, structural, and functional analyses of the entire human small-HERC family, which includes HERC3, HERC4, HERC5, and HERC6. We demonstrated that the *HERC* family emerged >595 million years ago and has undergone gene duplication and gene loss events throughout its evolution. The structural topology of the RCC1-like domain and HECT domains from all HERC paralogs is highly conserved among evolutionarily diverse vertebrates despite low sequence homology. Functional analyses showed that the human small HERCs exhibit different degrees of antiviral activity toward HIV-1 and that HERC5 provides the strongest inhibition. Notably, coelacanth HERC5 inhibited simian immunodeficiency virus (SIV), but not HIV-1, particle production, suggesting that the antiviral activity of HERC5 emerged over 413 million years ago and exhibits species- and virus-specific restriction. In addition, we showed that both HERC5 and HERC6 are evolving under strong positive selection, particularly blade 1 of the RCC1-like domain, which we showed is a key determinant of antiviral activity. These studies provide insight into the origin, evolution, and biological importance of the human restriction factor HERC5 and the other HERC family members.

**IMPORTANCE** Intrinsic immunity plays an important role as the first line of defense against viruses. Studying the origins, evolution, and functions of proteins responsible for effecting this defense will provide key information about virus-host relationships that can be exploited for future drug development. We showed that HERC5 is one such antiviral protein that belongs to an evolutionarily conserved family of HERCs with an ancient marine origin. Not all vertebrates possess all HERC members, suggesting that different HERCs emerged at different times during evolution to provide the host with a survival advantage. Consistent with this, two of the more recently emerged HERC members, HERC5 and HERC6, displayed strong signatures of having been involved in an ancient evolutionary battle with viruses. Our findings provide new insights into the evolutionary origin and function of the HERC family in vertebrate evolution, identifying HERC5 and possibly HERC6 as important effectors of intrinsic immunity in vertebrates.

## INTRODUCTION

Vertebrates possess multiple defense mechanisms to inhibit the replication of viruses. This defense system is largely composed of specialized hematopoietic cells that react nonspecifically to pathogens (innate immunity), an antibody-dependent and cell-mediated response (adaptive immunity), and core cellular effector proteins called restriction factors (intrinsic immunity). Restriction factors are considered to be the front line of defense against viral infection, since their activity typically does not require virus-triggered signaling or intercellular communication (1). The importance of intrinsic immunity in vertebrates is highlighted by the evolutionarily ancient origin and broad antiviral activity of restriction factors, such as bone marrow stromal antigen 2 (BST-2)/tetherin (2). Other restriction factors, such as apolipoprotein B mRNA-editing enzyme catalytic polypeptide-like 3G (APOBEC3G) and tripartite motif protein 5 alpha (TRIM5α), are unique to placental mammals and appear to play more specialized antiviral roles by targeting a more limited range of viruses, largely retroviruses (3–8).

Interferon-stimulated gene 15 (ISG15) and/or its conjugation to newly translated proteins (referred to as ISGylation) exhibits broad antiviral activity toward evolutionarily diverse viruses, including those belonging to the families Retroviridae, Orthomyxoviridae, Flaviviridae, Togaviridae, *Herpesviridae*, *Poxviridae*, Arteriviridae, and *Pneumoviridae* (9–31). The main cellular E3 ligase responsible for ISGylation activity is “homologous to the E6-AP carboxyl terminus (HECT) and regulator of chromosome condensation 1 (RCC1)-like domain-containing protein 5” (HERC5), an interferon (IFN)-induced restriction factor that has evolved under strong positive selection in vertebrates (11, 28–30, 32–36). HERC5 belongs to a subfamily of four small HERC proteins, HERC3 to -6. Although referred to as “small,” the small HERC proteins are ∼116 kDa in size, each containing a single amino-terminal RCC1-like domain and a carboxyl-terminal HECT domain. The small HERCs are classified as E3 ligases due to the presence of their HECT domains and their ability to conjugate ubiquitin or ubiquitin-like molecules to proteins (32–34). Although the biological functions of the small-HERC family have not been fully defined, their E3 ligase activities have been implicated in a variety of biological processes, such as protein degradation, cell signaling, spermatogenesis, tumor suppression, and antiviral defense (reviewed in reference 37).

By virtue of their RCC1-like domains, HERCs also belong to the phylogenetically widespread RCC1 superfamily of proteins (38, 39). The prototypical member of this superfamily is RCC1, characterized by the presence of seven repeats of 51 to 68 amino acids that assume a 7-bladed β-propeller structure. RCC1 is localized in the nuclei of eukaryotic cells and activates the GTPase Ras-related nuclear (Ran) protein (40). RCC1 maintains a >1,000-fold higher level of RanGTP in the nucleus than in the cytoplasm, which is critical for Crm1-dependent nuclear export of macromolecules (41, 42). We previously showed that human HERC5 inhibits the Crm1-dependent nuclear export of incompletely spliced human immunodeficiency virus type 1 (HIV-1) RNA, resulting in a severe reduction in the level of intracellular HIV-1 Gag protein and production of virus (29). This mechanism is independent of its E3 ligase activity. Blade 1 of the 7-bladed β-propeller RCC1-like domain structure of HERC5 was critical for this inhibition and contained numerous residues predicted to be evolving under positive selection, identifying the region as a potential key antiviral interface between HERC5 and viruses (29). Thus far, antiviral activity has been demonstrated only for human HERC5 and its functional homolog in mice, HERC6 (11, 28, 30, 32–36). Here, we investigate the evolutionary origins and antiviral activities of the HERC family members, providing a better understanding of the role these proteins play in intrinsic immunity.

## RESULTS

### The small-*HERC* gene family has an ancient marine origin more than 595 million years ago.

**FIG 1:**
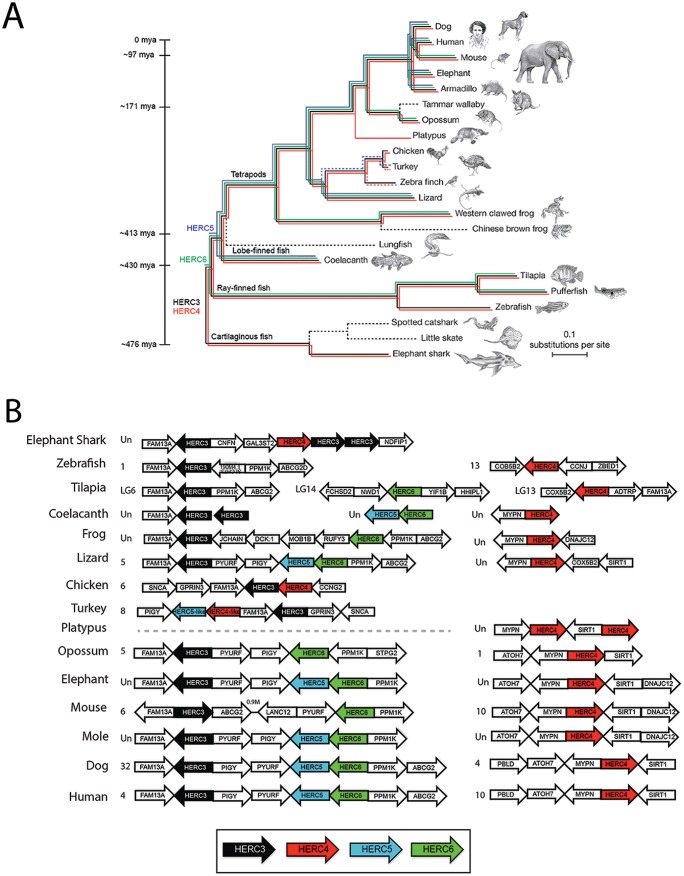
Emergence of the small-*HERC* family. (A) *HERC3* (black), *HERC4* (red), *HERC5* (blue), and *HERC6* (green) sequences were searched in genome assemblies using the UCSC Genome Browser (https://genome.ucsc.edu) and NCBI gene and protein sequence databases. The presence of *HERC* orthologs in species is indicated by colored lines. The approximate dates of divergence among the organisms are indicated by the time line on the left from the perspective of humans, as previously described by Hedges et al. (43). The Bayesian tree was obtained from a multiple-sequence alignment of 251 genes with a 1:1 ratio of orthologs in 22 vertebrates, rooted on cartilaginous fish (support was 100% for all clades but armadillo and elephant, with 45%), as described previously (45). The dashed black lines indicate that no *HERC* orthologs were identified in the species. The dashed red and blue lines indicate the presence of partial *HERC4*-like and *HERC5*-like sequences, respectively. (Adapted from reference 45 with permission of the publisher [Springer Nature].) (B) Syntenic relationships of the genomic contexts of the small-*HERC* loci in evolutionarily diverse vertebrates. Chromosome numbers are indicated on the left of each locus. Un, unplaced scaffold.

With the sequencing of many evolutionarily diverse vertebrate and mammalian genomes, we can approximate the emergence and divergence of gene families throughout evolution. We analyzed the most recent genome assemblies (UCSC Genome Browser [https://genome.ucsc.edu]) and NCBI gene and protein sequence databases for the presence of small *HERC* gene members. The oldest small-*HERC* member is *HERC4*, which is present in one of the only surviving lineages of jawless fish, sea lampreys (originating ∼595 million years ago [mya]) (43). To better understand the evolution of the small-*HERC* family, we investigated the emergence and divergence of *HERC* genes in evolutionarily diverse vertebrates. The elephant shark is among the oldest and most slowly evolving jawed vertebrates and has accumulated a small number of chromosomal rearrangements (44). This allowed us to look for evidence of gene expansion at an early point in vertebrate evolution (∼476 mya) (43). A single copy of *HERC4* and multiple copies of *HERC3* are present in elephant sharks. Two copies of *HERC3* are located immediately adjacent to *HERC4*, likely representing an early point in vertebrate evolution (∼476 to 595 mya), just after the small-*HERC* family expanded with the duplication and divergence of *HERC4* (Fig. 1). *HERC3* and *HERC4* are present in all the jawed vertebrates examined except the platypus, which appears to be the only vertebrate that contains two copies of *HERC4*. Since the only available assembly for platypus is considered low coverage, future improvements in the assembly are needed to help explain this apparently unique composition of the *HERC* family in these mammals. Chromosomal rearrangement likely occurred sometime after the divergence of ray-finned fish from cartilaginous fish (∼430 mya), giving rise to two different chromosomal *HERC* loci in most vertebrates, where the *HERC3-HERC5-HERC6* locus is flanked by *FAM13A* and *PIGY-PYURF* and *HERC4* by *MYPN* and *SIRT1* (Fig. 1B).

The *HERC* family expanded further after the divergence of cartilaginous fish (∼430 mya), with the emergence of *HERC6*, which is present in most jawed vertebrates with an apparent absence in platypus and some fish (e.g., zebrafish) and bird (e.g., chicken, turkey, and zebra finch) species (Fig. 1). The last expansion of the *HERC* family occurred after the divergence of ray-finned fish (∼413 mya), with the likely duplication of *HERC6*, giving rise to *HERC5* (Fig. 1B). *HERC5* is present in the coelacanth, one of the earliest predecessors of tetrapods, and appears to have been lost in some species of frogs, birds (e.g., chicken), metatherian (marsupial) mammals (e.g., opossum), and rodents (e.g., mouse) (Fig. 1) (43, 45). A partial *HERC5*-like gene was identified in turkey, and a partial *HERC4*-like gene was identified in turkey and finch, possibly indicating erosion of the gene family in birds. In species that appear to have lost *HERC* orthologs, we cannot rule out the possibility that the orthologs are present but were missed due to low sequence homology and/or incomplete genome annotation. Together, these findings indicate that the small-*HERC* family has an ancient marine origin at least 595 mya, before the emergence of jawed vertebrates, and has undergone chromosomal rearrangement, gene duplication, and potential gene loss events during vertebrate evolution.

### Evolutionarily distant RCC1-like domains and HECT domains are well conserved.

**FIG 2:**
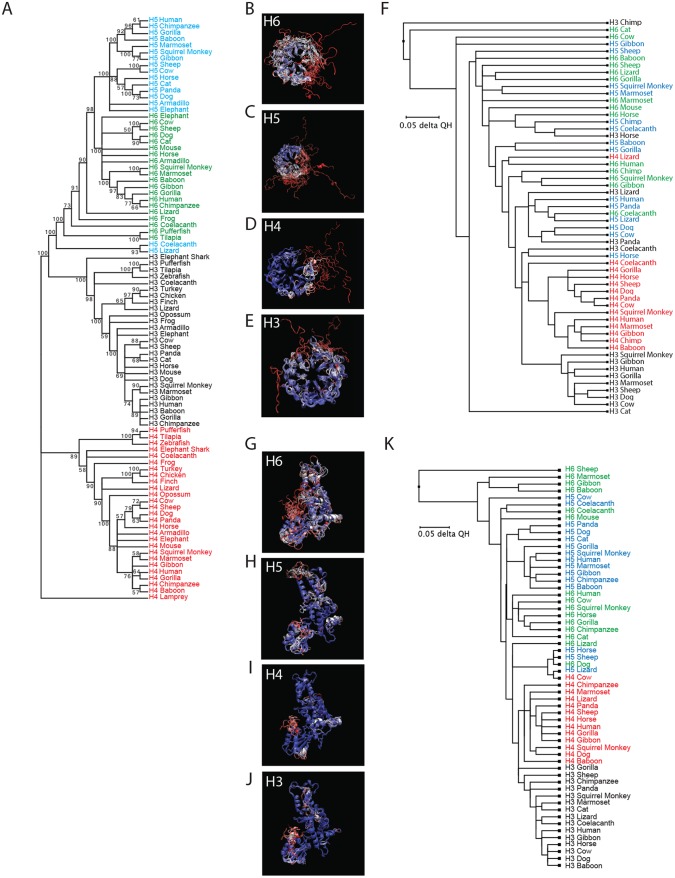
(A) Molecular phylogenetic analysis using the maximum likelihood method. The evolutionary history was inferred by using the maximum likelihood method based on the JTT matrix-based model (89). The bootstrap consensus tree, inferred from 100 replicates, was taken to represent the evolutionary histories of the taxa analyzed. Branches corresponding to partitions reproduced in less than 50% of the bootstrap replicates were collapsed. The percentages of replicate trees in which the associated taxa clustered together in the bootstrap test (100 replicates) are shown next to the branches. Initial trees for the heuristic search were obtained automatically by applying neighbor-joining and BioNJ algorithms to a matrix of pairwise distances estimated using a JTT model and then selecting the topology with a superior log likelihood value. The analysis involved 91 amino acid sequences. All positions containing gaps and missing data were removed. There were a total of 433 positions in the final data set. Evolutionary analyses were conducted in MEGA7 (83). H3, HERC3; H4, HERC4; H5, HERC5; H6 HERC6. (B to F) Evolutionary conservation of HERC RCC1-like domains. (B to E) Predicted structures of the RCC1-like domains were generated using 3D-Jigsaw (v2.0). Multiple structural alignments were generated based on the Q_H_ structural measure using the program STAMP, a plug-in in the MultiSeq interface of the Visual Molecular Dynamics (VMD) software (v1.9.2). (F) Three-dimensional representation of the structural data colored by structural conservation. Each amino acid is colored according to the degree of conservation within the alignment: blue, highly conserved; white, somewhat conserved; and red, very low or no conservation. The structure-based cladogram was derived from sequence and structural alignments of the predicted tertiary structures of multiple mammalian HERC RCC1-like domains. (G to K) Evolutionary conservation of HERC HECT domains. (G to J) Three-dimensional representations of the predicted HECT domain structural data colored by structural conservation. (K) Structure-based cladogram derived from sequence and structural alignments of the predicted tertiary structures of multiple mammalian HERC HECT domains. See Table S1 in the supplemental material for the parent structure scaffolds used to generate all the models.

Phylogenetic analysis of *HERC* sequences showed segregation of the small *HERC* genes into four major clusters consisting of *HERC3*, *HERC4*, *HERC5*, and *HERC6* (Fig. 2A). Most *HERC* orthologs have high sequence homology, ranging from ∼70 to 100% amino acid identity, whereas most *HERC* paralogs have low homology, ranging from ∼34 to 57% (see Fig. S1 in the supplemental material). Notably, coelacanth and lizard *HERC5* sequences clustered on their own, showing more sequence similarity to *HERC6* genes than to other *HERC5* genes, possibly indicating that these genes are actually *HERC6* or a hybrid of *HERC5* and *HERC6*. Similar tree topologies regarding the main branching were predicted using several tree-generating algorithms (maximum likelihood, minimal evolution, unweighted pair group method using average linkages [UPMGA], and neighbor-joining methods). Consistent with the approximate emergence times of the small-*HERC* family members shown in Fig. 1, *HERC4* is the oldest of the *HERC* paralogs, followed by *HERC3*, *HERC6*, and then *HERC5*.

The small HERCs are believed to have arisen from a gene fusion event between an RCC1-like domain and a HECT domain (46). Although the approximate date of this event is unknown, the presence of HERC4 in jawless fishes (e.g., lampreys) suggests that the fusion event occurred more than 595 mya. Typically, the primary amino acid sequences of RCC1-like domains have low sequence homology in the RCC1 superfamily; however, their tertiary structures are highly conserved (38, 39). To assess how conserved the predicted tertiary structures are among the different small-HERC members, we generated a phylogenetic tree based on the Q_H_ structural measure derived from alignment of the predicted tertiary structures of RCC1-like domains (Fig. 2B to F; see Table S1 in the supplemental material). The models were predicted using 3D-Jigsaw v2.0 (https://bmm.crick.ac.uk/~populus/) and showed that each of the RCC1-like domains of HERC3 to -6 adopted the characteristic β-propeller structure of the superfamily, despite their low sequence homology (47–51). Alignment of the structures was carried out using the program Structural Alignment of Multiple Proteins (STAMP), which is a tool for aligning protein sequences based on their three-dimensional structures (52). The STAMP algorithm minimizes the Cα distance between aligned residues of each molecule by applying globally optimal rigid-body rotations and translations. STAMP analysis revealed that the RCC1-like domains of the HERC orthologs are well conserved overall, with HERC3 and HERC4 being the most conserved (Fig. 2B to F). Several paralogs showed greater similarity to each other than to their orthologous counterparts (e.g., lizard HERC4 and human HERC6). Chimpanzee HERC3 differed substantially from the other HERCs in that it lacks blade 3 of the β-propeller. Notably, the amino-terminal blade 1 of each RCC1-like domain is the least conserved region and adopts a more extended conformation than the other blades.

Analysis of the predicted HECT domain structures showed that they all adopted the typical bilobal structure of HECT domains (Fig. 2G to K). STAMP analysis showed that the different orthologs are well conserved overall, with HERC3 and HERC4 being the most conserved. Some HERC5 and HERC6 paralogs shared more similarity to each other than to their respective orthologs. Notably, coelacanth and mouse HERC6 proteins were more similar to HERC5 paralogs than to other HERC6 orthologs, potentially indicating conservation in structure and function of these HECT domains (Fig. 2K). This is consistent with the finding that mouse HERC6 is the functional homolog of human HERC5; these are the main cellular E3 ligases for ISG15 in mice and humans, respectively. Together, these data show that despite low sequence homology, evolutionarily divergent *HERC* genes share remarkable similarity in the predicted structures of their RCC1-like domains and HECT domains.

### Human HERC3 to -6 differentially inhibit HIV-1 particle production.

**FIG 3:**
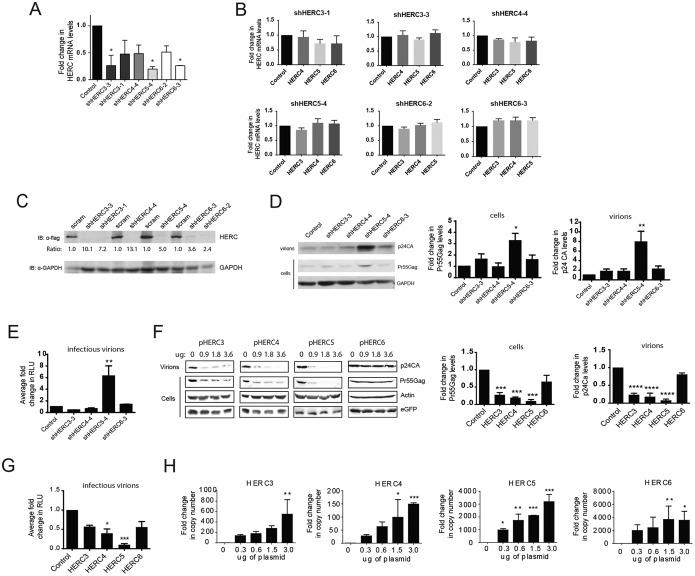
HERC3 to -6 differentially restrict HIV-1 particle production. (A and B) HOS-CD4-CXCR4 cells were transfected with a plasmid carrying either scrambled shRNA (control) or shRNAs to each of the different HERCs independently. HERC mRNA levels were measured by qPCR using HERC-specific primers. The average fold changes in HERC mRNA levels (with standard errors of the mean [SEM]) from the results of 3 independent experiments are shown. One-way analysis of variance (ANOVA) and Dunnett's multiple-comparison test with the control were performed. (C) 293T cells were first transfected with plasmids carrying HERC shRNA and 24 h later with the respective Flag-tagged HERC constructs. HERC protein levels were measured by quantitative Western blotting (immunoblotting [IB]) using anti (α)-Flag or anti-GAPDH (loading control). The numbers below the blots represent the average fold changes in HERC protein levels compared to the control cells after densitometric quantification of the Flag-tagged HERC bands. scram, scrambled shRNA. (D) HOS-CD4-CXCR4 cells were transfected with pR9 and plasmids carrying either scrambled shRNA (control) or shRNAs to each of the different HERCs independently. Seventy-two hours after transfection, cell lysates and HIV-1 virions in the supernatant were harvested. HIV-1 particle production was measured by quantitative Western blotting using anti-p24CA or anti-GAPDH. (E) Average (plus SEM) densitometric quantifications of Pr55Gag (cells) and p24CA (virions) bands. Virions produced from the cells were used to infect the luciferase reporter cell line TZM-bl to measure the number of infectious virions. The average fold changes in relative light units (RLU) from the results of at least 3 independent experiments are shown. (F and G) 293T cells were cotransfected with pR9 and peGFP (transfection control) and increasing amounts of either empty vector, pHERC3, pHERC4, pHERC5, or pHERC6. Virions in the supernatant and total cell lysates were subjected to quantitative Western blot analysis using anti-p24CA, anti-eGFP, and anti-β-actin as a loading control. The average (plus SEM) densitometric quantification of p24CA and Pr55Gag levels from Western blot images of virions or cell lysate (3.6 μg HERC lane) are shown on the right. The values were normalized to β-actin and eGFP levels. (G) Virions produced from cells were used to infect the luciferase reporter cell line TZM-bl to measure the number of infectious virions. (H) 293T cells were transfected with either empty vector or increasing amounts of plasmids encoding the different HERCs. Twenty hours posttransfection, total RNA was harvested from the cells and subjected to qPCR to measure HERC mRNA levels. Relative fold changes in HERC mRNA compared to the control cells are shown. Statistical significance was determined using one-way ANOVA and Dunnett's multiple-comparison test with the control cells. *, *P* < 0.05; **, *P* < 0.01; ***, *P* < 0.001; ****, *P* < 0.0001.

Given the remarkable similarity in the predicted structures of the HECT and RCC1-like domains, we asked whether human HERC3, HERC4, and HERC6 inhibited HIV-1 particle production like HERC5. To test the effect on HIV-1 replication of knocking down endogenous HERC protein levels, we first screened different HERC short hairpin RNA (shRNA) constructs for the ability to knock down endogenous HERC mRNA and protein levels. As shown in Fig. 3A, several of the shRNA constructs knocked down HERC mRNA levels by 2- to 5-fold. For each shRNA construct used, no significant differences in mRNA levels were detected for any of the other related small HERCs, demonstrating specificity (Fig. 3B). Unfortunately, we were unable to readily measure endogenous levels of HERC proteins using several different commercially available antibodies. This could be due to poor recognition of endogenous HERC proteins by the antibodies and/or tightly controlled cytosolic levels of HERC proteins, as previously observed (34). As such, HERC protein knockdown efficiencies of the shRNA constructs were instead determined using exogenously expressed Flag-tagged HERC constructs (Fig. 3C). ShRNAs that knocked down Flag-tagged HERC3, HERC4, HERC5, and HERC6 protein levels by 10.1-, 7.2-, 5.0-, and 3.6-fold, respectively, were used for subsequent experiments.

HOS-CD4-CXCR4 cells were cotransfected with plasmids carrying HERC shRNA and HIV-1 R9 (a full-length replication-competent NL4-3 derivative). After 72 h of replication (∼2 or 3 rounds), quantitative Western blot analysis of cell lysates or virions produced from cells showed that cells knocked down for HERC5 expression exhibited a significant increase in Gag particle production (∼8-fold), whereas HERC3, -4, and -6 released modestly more virions into the supernatant than the control cells (∼2-fold) (Fig. 3D). The amount of infectious HIV-1 in the supernatant was also measured by infecting the TZM-bl indicator cell line, which enabled quantitative analysis of HIV-1 using luciferase as a reporter (53–57). Cells knocked down for endogenous HERC5 expression failed to inhibit production of infectious HIV-1, whereas cells knocked down for HERC3, HERC4, or HERC6 expression released levels of infectious virions similar to those of the control cells (Fig. 3E).

To test the effect of increased HERC expression on single-round HIV-1 particle production, human 293T cells, which do not support multiround replication, were cotransfected with plasmids carrying HIV-1 (R9) and either empty vector, HERC3, HERC4, HERC5 or HERC6. As expected, HERC5 potently inhibited HIV-1 particle production (Fig. 3F). HERC3 or HERC4 also significantly inhibited particle production, but not as potently as HERC5. In contrast, HERC6 modestly inhibited HIV-1 particle production but did not achieve statistical significance. As expected, HERC5 also potently inhibited the production of infectious HIV-1, whereas HERC3, HERC4, and HERC6 modestly inhibited production of infectious HIV-1 (Fig. 3G). Each transfected HERC construct exhibited robust mRNA expression, although HERC3 and HERC4 levels were less than those of HERC5 and HERC6 (Fig. 3H). Taken together, these data show that upregulated expression of HERC3 to -6 inhibited HIV-1 particle production and replication to varying degrees, with HERC5 exhibiting the most potent activity and HERC6 the weakest activity. Notably, only endogenous levels of HERC5 significantly inhibited production of infectious HIV-1.

### Human HERC3 to -6 differentially inhibit nuclear export of incompletely spliced RNA.

**FIG 4:**
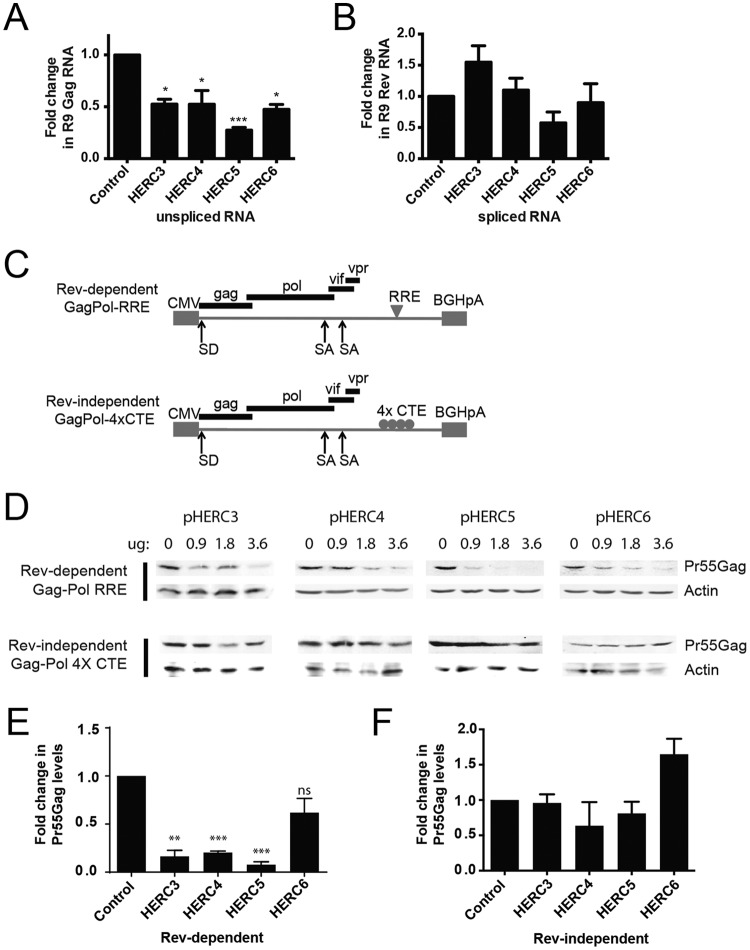
HERC3 to -5 inhibit cytoplasmic accumulation of unspliced HIV-1 RNA. (A and B) 293T cells were cotransfected with pR9 and peGFP (transfection control) and either empty vector, pHERC3, pHERC4, or pHERC5. Forty-eight hours after transfection, total RNA was extracted and reverse transcribed into cDNA from whole-cell lysates or from the cytoplasmic fraction only. Quantitative PCR was performed on each fraction using primers specific for unspliced HIV-1 genomic RNA (e.g., Gag), fully spliced RNA (e.g., Rev), β-actin (loading control), or eGFP (transfection control). The proportion of unspliced or fully spliced HIV-1 RNA in the cytoplasmic fraction compared to the total amount of HIV-1 RNA (nuclear plus cytoplasmic) was determined for control cells and cells expressing HERC after normalization to β-actin and eGFP levels. The fold changes in copy numbers relative to control cells are shown. The data shown represent the averages (plus SEM) from the results of four independent experiments. (C) Schematic depicting the different Gag-Pol constructs used in the experiment shown in panel D. CMV, cytomegalovirus; SA, splice acceptor; SD, splice donor. (D) 293T cells were cotransfected with increasing amounts of plasmids encoding HERC3, HERC4, or HERC5 and either Rev-dependent GagPol-RRE (plus pRev) or Rev-independent GagPol-4×CTE. The total DNA transfected was kept equal with the empty-vector plasmid. Gag levels within the cell lysates were analyzed by quantitative Western blotting using anti-p24CA and anti-β-actin as a loading control. (E and F) Densitometric quantification of Pr55Gag bands from the lanes containing the largest amount of each HERC in the Western blots from panel D was performed. Shown are the average fold changes (plus SEM) in Pr55Gag levels relative to the empty-vector control after normalization to β-actin levels. Statistical significance was determined using ANOVA with Dunnett's multiple-comparison test. *, *P* < 0.05; **, *P* < 0.01, ***, *P* < 0.001; ns, not significant.

We previously showed that human HERC5 blocked nuclear export of Rev-dependent HIV-1 RNA (29). To determine if HERC3, HERC4, and HERC6 also blocked nuclear export of Rev-dependent HIV-1 RNAs, we cotransfected 293T cells with plasmids carrying full-length HIV-1 R9 and either empty vector, HERC3, HERC4, HERC5, or HERC6. A plasmid encoding enhanced green fluorescent protein (eGFP) was also cotransfected to serve as a transfection control. Total RNA was harvested from the total cell extract or the cytoplasmic extract only and subjected to quantitative PCR (qPCR) with primers specific for either Gag (unspliced HIV-1 genomic RNA), Rev (fully spliced RNA), β-actin (loading control), or eGFP. Each of the small HERC proteins exhibited significant reductions in the amount of HIV-1 genomic RNA present in the cytoplasm, with HERC5 exhibiting the strongest activity (Fig. 4A). In contrast, no significant reductions in the export of fully spliced HIV-1 transcripts were observed (Fig. 4B).

To further support this finding, we tested the abilities of the small HERCs to inhibit Gag expression from Rev-dependent (e.g., GagPol-RRE) and Rev-independent (e.g., GagPol-4×CTE) constructs, as previously described (29, 58). HIV-1 Rev promotes nuclear export of incompletely spliced HIV-1 mRNAs by binding to a specific *cis*-acting element called the Rev-response element (RRE) located within an HIV-1 intron (Fig. 4C). HIV-1 mRNA containing four copies of the Mason-Pfizer monkey virus constitutive export element (4×CTE) in place of the RRE is not dependent on Rev for nuclear export and thus serves as a Rev-independent control (59). Successful export of incompletely spliced RNA can be assessed by Western blotting for Gag protein expression. 293T cells were cotransfected with a plasmid encoding Rev and increasing concentrations of plasmids encoding HERC, with or without pGagPol-RRE or pGagPol-4×CTE. As shown in Fig. 4D and E, each of the small HERCs differentially inhibited nuclear export of Rev-dependent RNA, where HERC5 exhibited the highest level of inhibition and HERC6 the weakest inhibition. In contrast, none of the HERCs significantly inhibited nuclear export of Rev-independent RNA (Fig. 4D and F). Together, these findings indicate that the small-HERC members differentially inhibit nuclear export of Rev-dependent RNAs.

### Antiviral activity of HERC5 evolved more than 413 million years ago.

**FIG 5:**
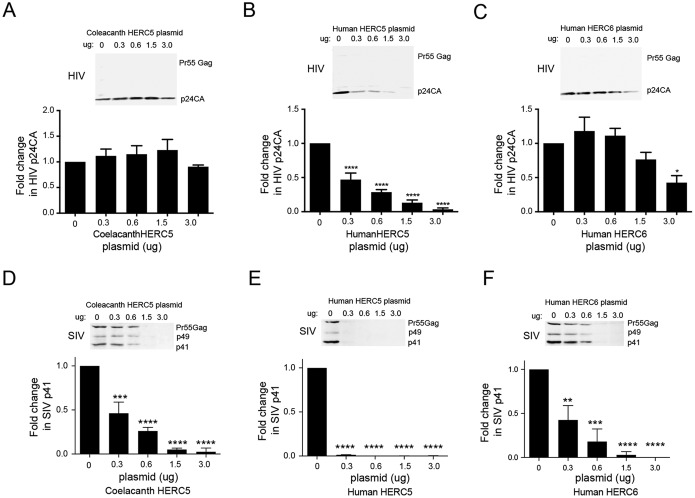
Coelacanth HERC5 restricts SIV, but not HIV-1, particle production. 293T cells were cotransfected with a plasmid carrying HIV-1 (pR9) (A to C) or SIV (pSIVmac239) (D to F) and increasing amounts of plasmids encoding either coelacanth HERC5, human HERC5, or human HERC6. Forty-eight hours posttransfection, virus released into the supernatant was measured by quantitative Western blotting of Gag proteins using monoclonal anti-p24CA (183-H12-5C) or anti-SIVp17 (KK59). The average relative fold changes (plus SEM) in HIV p24CA or SIV p24CA protein levels compared to the control cells after densitometric quantification of 3 independent Western blot images are shown. Statistical significance was determined using one-way ANOVA with Dunnett's multiple-comparison test with the control cells. **, *P* < 0.01; ***, *P* < 0.001; ****, *P* < 0.0001.

We next asked whether the antiretroviral activity of human HERC5 has an evolutionarily ancient origin. Since the coelacanth was the oldest vertebrate in which we identified *HERC5*, we tested the ability of coelacanth HERC5 to inhibit HIV-1 virus production. To assess potential virus-specific effects, we also tested the antiviral activity toward another, related nonhuman retrovirus, simian immunodeficiency virus (SIV) (SIVmac239, a full-length rhesus macaque derivative lacking the 5′ long terminal repeat [LTR]), which is thought to be at least 32,000 years older than HIV-1 (60). For comparison, we included human HERC5 and human HERC6, which exhibited the strongest and weakest anti-HIV-1 activities, respectively. Human 293T cells were cotransfected with plasmids carrying SIVmac239 or HIV-1 R9 and increasing concentrations of either empty vector, coelacanth HERC5, human HERC5, or human HERC6. Forty-eight hours after transfection, virus released into the supernatant was measured by Western blotting. As expected, human HERC5 exhibited strong inhibition, whereas both coelacanth HERC5 and human HERC6 exhibited little inhibition of HIV-1 (Fig. 5A to C). In contrast, each of the HERCs inhibited SIVmac239 virus production, with human HERC5 being the most potent (Fig. 5D to F). Together, these results demonstrate that the antiretroviral activity of HERC5 has an ancient marine origin at least 413 mya and that HERC5 and HERC6 exhibit species- and virus-specific antiviral activity.

### HERC6 is evolving under positive selection.

We previously showed that HERC5, especially blade 1 of its RCC1-like domain, has been evolving under positive selection for >100 million years (29). We performed a similar analysis for each of the small-HERC members to determine if the other members of the small-HERC family have been evolving under positive selection. HERC evolution in mammals was evaluated under several standard models of sequence evolution using the Server for the Identification of Site-Specific Positive Selection and Purifying Selection (Selecton) program (61–64). This comprised two nested pairs of models (M8a and M8; M7 and M8), in which the first model of each pair is nested in the second model. The M8 model, but not the M8a or M7 model, allows sites to evolve under positive selection. A nonnested-pair (M8a and MEC) model comparison was also performed. The MEC model differs from the other models in that it takes into account the differences between amino acid replacement rates (61). The nested models were compared using the likelihood ratio test.

**FIG 6:**
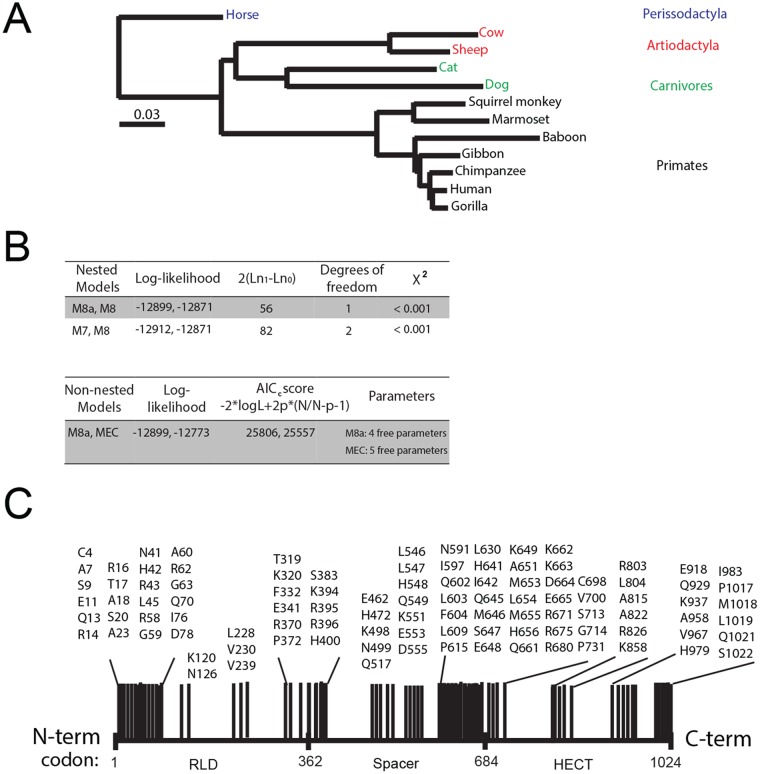
*HERC6* is evolving under strong positive evolutionary selection. (A) Neighbor-joining phylogenetic tree for progressive alignment of 12 different *HERC6* species using COBALT for multiple protein sequences. The branch lengths are proportional to the amount of inferred evolutionary change. (B) Analysis for positive selection was performed using HERC6 amino acid sequences from human (Homo sapiens), chimpanzee (Pan troglodytes), gorilla (Gorilla gorilla gorilla), marmoset (Callithrix jacchus), baboon (Papio anubis), squirrel monkey (*Saimiri boliviensis*), gibbon (Nomascus leucogenys), horse (Equus caballus), sheep (Ovis aries), cow (Bos taurus), dog (Canis lupus familiaris), and cat (Felis catus). Evolutionary analysis for positive selection in *HERC6* used various models of evolution, where M8 and MEC allowed sites to evolve under positive selection and M7 and M8a did not. L represents the likelihood of the model given the data, p represents the number of free parameters, and N represents the sequence length. The lower the AIC_C_ score, the better the fit of the model to the data, and hence, the model is considered more justified. (C) Schematic showing the results of a Bayesian analysis approach identifying positively selected sites with a ratio of the number of nonsynonymous substitutions per nonsynonymous site (Ka) to the number of synonymous substitutions per synonymous site (Ks) (Ka/Ks) with a value of >1.5 and a 95% confidence interval larger than 1 and therefore considered statistically significant. The *HERC6* reference sequence accession number is NM_017912.3.

Analysis of 12 evolutionarily diverse HERC sequences using Selecton revealed that HERC6, but not HERC3 and HERC4, is evolving under positive selection (Fig. 6; see Table S2 in the supplemental material). Allowing sites to evolve under positive selection (M8) gave a significantly better fit to the HERC6 sequence data than the corresponding model without positive selection (M8a and M7) (Fig. 6B). The MEC model, which allows positive selection, was compared with the M8a null model, which does not allow positive selection. Comparison of the Akaike Information Criterion (AICc) scores (M8a, 25,806; MEC, 25,557) revealed that the MEC model fits the HERC6 data better than the M8a model. The results of the MEC analysis were projected onto the primary sequence of human HERC6 (Fig. 6C). Notably, ∼23% (23 of 102) of the codons cluster within the first 80 amino acids of the amino terminus of the RCC1-like domain, encompassing blade 1 of its predicted β-propeller structure. Another ∼32% (33 of 102) cluster at the carboxyl terminus of the spacer region (amino acids ∼630 to 680). These results show that strong positive selection is operating on HERC6, with a large number of codons in blade 1 of the RCC1-like domain and the carboxyl terminus of the spacer region evolving under positive selection.

### Blade 1 of the RCC1-like domain of human HERC6 is an important determinant of anti-HIV-1 activity.

**FIG 7:**
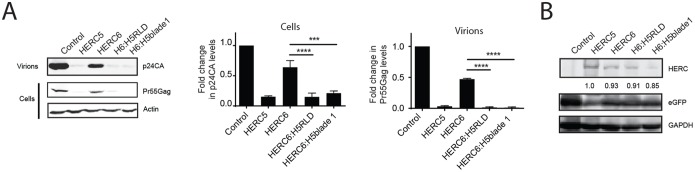
Blade 1 of the human HERC5 RCC1-like domain confers potent antiviral activity on human HERC6. (A) 293T cells were cotransfected with pR9, peGFP, and either empty vector, pHERC5, pHERC6, pH 6:H5RLD, or pH6:H5blade1. Forty-eight hours posttransfection, virions in the supernatant and total cell lysates were subjected to quantitative Western blot analysis using anti-p24CA or anti-β-actin. Densitometric quantification of p24CA (virions) and Pr55Gag (cells) from the results of three independent experiments is shown beside the blots after normalization to the β-actin loading controls. The error bars indicate SEM. ***, *P* < 0.001; ****, *P* < 0.0001. (B) Cell lysates from panel A were subjected to Western blot analysis using anti-Flag, anti-eGFP, or anti-GAPDH. Average densitometric quantification of the HERC bands is shown below the blot after normalization to anti-eGFP or anti-GAPDH from the results of three independent experiments.

Given the evolutionary similarities between human HERC5 and human HERC6, we asked why they differed in their antiviral activities. Since we previously showed that blade 1 of HERC5 is required for its anti-HIV-1 activity and that blades 1 of both HERC5 and HERC6 contain numerous residues evolving under positive selection, we asked if blade 1 of HERC5 can confer antiviral activity on HERC6. We replaced either the entire RCC1-like domain (H6:H5RLD) or blade 1 (H6:H5blade1) from human HERC5 with the corresponding region in human HERC6. We then measured the abilities of these HERC6 mutants to inhibit HIV-1 particle production. As shown in Fig. 7A, the H6:H5RLD and H6:H5blade1 mutants potently inhibited HIV-1 particle production similarly to wild-type HERC5. This inhibition occurred despite levels of H6:H5RLD and H6:H5blade1 protein expression slightly lower than that of wild-type HERC5 (Fig. 7B). This result indicates that blade 1 is an important determinant of antiviral activity.

## DISCUSSION

We showed here that the small-*HERC* family has an ancient marine origin, where *HERC4* emerged at least 595 mya and expansion of the family occurred sometime after the divergence of jawed vertebrates from jawless vertebrates (∼476 to 595 mya). Elephant sharks are among the oldest and most slowly evolving jawed vertebrates and have accumulated a small number of chromosomal rearrangements (44). Thus, analysis of their genome allows us to gain insight into the early evolution and expansion of gene families. The presence of a single copy of *HERC4* and multiple copies of *HERC3* in the elephant shark likely represents an early time in vertebrate evolution when the *HERC4* ancestral gene duplicated and evolved into *HERC3*. Although the evolutionary pressures in vertebrates triggering expansion of the small-*HERC* family are unknown, it is plausible, given their antiretroviral activity, that this expansion involved retroviruses. Endogenous retroviruses (ERVs) comprise a substantial portion of vertebrate genomes and appear to have an ancient marine origin, with evidence of ERV sequences found in the genomes of elephant shark, coelacanth, and possibly lamprey (65–68). A recent panvertebrate comparative genomic analysis showed that retroviruses have an unprecedented capacity for rampant host switching among distantly related vertebrates, undoubtedly exerting substantial evolutionary pressure on their hosts (68). Pressures like these can trigger antiviral gene duplication and neofunctionalization events in the hosts, allowing them to evolve more rapidly in order to maintain evolutionary dominance over viruses. Several examples where gene duplication/neofunctionalization has given rise to restriction factor families in primates are *MX1*, *IFITM*, *TRIM5*, and *APOBEC3* (5, 6, 69–74). These genes, including *HERC5* and *HERC6*, exhibit strong signatures of positive selection, which is consistent with repeated exposure to such evolutionary pressures.

An interesting feature of the small-HERC family is the highly conserved topology of the RCC1-like domain, despite limited sequence homology. By allowing numerous amino acid substitutions while maintaining the overall protein configuration and antiviral activity, these HERC proteins may be able to interfere with the binding of diverse viral antagonists. Evidence of such an evolutionary battle lies in the strong signatures of positive selection in both human HERC5 and HERC6, especially blades 1 of their RCC1-like domains, which we have shown to be important determinants of antiviral activity. HERCs are not the only restriction factors likely to have played an important antiviral role early in vertebrate evolution. BST2/tetherin has also been shown to have an ancient marine origin, emerging >450 mya, before the separation of cartilaginous fish from bony vertebrates (2). Like the HERC family, the general topology of BST2/tetherin orthologs are also highly conserved despite low sequence homology, and it is the overall protein configuration that is important for its antiviral activity (2, 75, 76). This evolutionary strategy may help BST2/tetherin and HERC proteins maintain evolutionary dominance over viruses.

We observed that some small *HERC* genes have been lost in some species, most notably birds and rodents. For birds, this is not too surprising, given that their genomes have been subjected to lineage-specific erosion of repetitive elements, large segmental deletions, and gene loss (>1,000 genes), resulting in a smaller repertoire of immune genes than in humans (77, 78). Although *HERC5* and *HERC6* are missing in most bird species, *HERC3* and/or *HERC4* is present. Given their modest antiretroviral activity in humans, it will be interesting to learn if HERC3 and HERC4 play an antiviral role in birds, perhaps compensating for the loss of *HERC5* and *HERC6*. Other potent antiviral restriction factor genes, such as *BST2/tetherin*, *TRIM22*, *TRIM5*, and *APOBEC3G*, are also notably absent from birds; however, they do possess other members of the *BST*, *TRIM*, and *APOBEC* families, whose antiviral activities remain largely uncharacterized in birds. Rodents also have *HERC3* and *HERC4* but also possess at least one of the *HERC5* and *HERC6* genes. For example, mice, rats, and hamsters possess *HERC6* but lack *HERC5*, ground squirrels possess *HERC5* but lack *HERC6*, and guinea pigs possess both *HERC5* and *HERC6*. Since no rodent lacks both *HERC5* and *HERC6*, it is likely that one of these genes has assumed the role of the main cellular E3 ligase for ISG15 in the absence of the other, potentially adding a new antiviral defense for rodents. For instance, this is the case in mice, which possess only *HERC6*, which encodes the main cellular E3 ligase for ISG15 and serves as a critical antiviral defense mechanism in mice (79, 80). Our phylogenetic analysis, where we showed that the predicted structures of the HECT domains of mouse HERC6 and human HERC5 (the main cellular E3 ligase for ISG15 in humans) share a high degree of similarity, also supports this finding. One possibility for the loss of *HERC5* or *HERC6* in rodents is that retroviruses or other viral pathogens have not provided constant selective pressure to maintain both genes in these species. A similar dynamic history of gene expansion and loss is evident for other restriction factors, such as *TRIM22*, *TRIM5*, and *BST2*/*tetherin* (2, 7, 81).

As genes duplicate, neofunctionalize, and diverge in response to evolutionary pressures, reliance on the activities of the ancestral genes may diminish, or they may be replaced altogether by their more advantageous descendants (reviewed in reference 82). Therefore, it is not surprising that the human small-HERC family exhibits differential antiviral activity. Despite still possessing antiviral activity when overexpressed *in vitro*, it is unlikely that HERC3 and HERC4 play significant biological roles as antiviral proteins in humans, since they are not IFN induced, nor have they been evolving under positive selection. This role was likely assumed by HERC5 and HERC6 after the divergence of ray-finned fish from cartilaginous fish (∼430 mya). However, *HERC3* and *HERC4* do exhibit differential tissue-specific expression (reviewed in reference 37). Therefore, it is possible that HERC3 and HERC4 play more dominant antiviral roles in tissues where their basal expression is already much more elevated, such as in the brain, heart, and stomach for HERC3 and the brain, lung, and testis for HERC4. Moreover, species such as elephant shark, coelacanth, and platypus that contain duplicated copies of *HERC3* or *HERC4* genes may also express higher levels of these HERC proteins due to increased gene copy numbers. Although we did not test the antiviral activities of other evolutionarily diverse HERC3 or HERC4 proteins, our phylogenetic analyses demonstrated that the HECT and RCC1-like domains of these proteins show remarkable structural similarity to their human counterparts, which do exhibit antiviral activity at elevated levels. It will be interesting to determine if the antiviral function of HERC3 and HERC4 is conserved in these ancient vertebrates, before the emergence of HERC5 and HERC6.

Interactions between host antiviral proteins and their viral-protein targets can be a critical requirement for their antiviral activity. When viral proteins mutate to evade such interactions, the antiviral protein frequently develops rapid amino acid replacements at the protein-protein interface in an attempt to restore those interactions and maintain evolutionary dominance over the virus. HERC5 is known to interact with evolutionarily diverse viral proteins and, like HERC6, is evolving under strong positive selection (11, 28–30, 32, 35, 36). Therefore, it is highly likely that these proteins contain one or more protein-protein interaction interfaces between viral and host proteins. Our findings that blades 1 of the RCC1-like domains of HERC5 and HERC6 contain numerous positively selected residues and that these residues are important determinants of antiretroviral activity indicate that blade 1 is likely one such interface. Although there is currently no evidence that retroviruses have driven positive selection of blade 1, it is interesting that blade 1 of HERC5 is sufficient to confer antiretroviral activity on HERC6. It is possible that the topology of blade 1 from HERC5 is such that it promotes interaction with a cellular protein required for activity that blade 1 of HERC6 prevents. However, our finding that wild-type HERC6 potently inhibited SIVmac239, but not HIV-1, in the same cell type suggests that virus-specific differences are more likely to account for the observed differential antiviral activity between HERC5 and HERC6. Additional structure-function studies are needed to differentiate between these possibilities and others.

In conclusion, the small-HERC family has an evolutionarily ancient origin more than 595 mya, with the latest expansion of the family occurring more than 413 mya. We showed that the structural topologies of the HECT and RCC1-like domains are highly conserved despite low sequence homology and that the antiretroviral activity of HERC5 has an ancient marine origin. HERC5 and HERC6 are evolving under strong positive selection, and a patch of positively selected residues in blade 1 of the RCC1-like domain is a strong determinant of antiviral activity. Altogether, our study highlights the potential importance of the HERC family in intrinsic immunity.

## MATERIALS AND METHODS

### Cell lines.

293T cells were obtained from the American Type Culture Collection. HOS-CD4-CXCR4 and TZM-bl cells were from the NIH AIDS Reagent Program. The cells were maintained in standard growth medium (Dulbecco's modified Eagle's medium [DMEM]) supplemented with 10% heat-inactivated fetal bovine serum (FBS), 100 U/ml penicillin, and 100 μg/ml streptomycin at 37°C with 5% CO_2_.

### Analyses of sequences and synteny.

The MEGA 7.0 package was used for phylogenetic analysis (83). The amino-terminal end of the small HERCs varies in length among the different members and was not included for phylogenetic analysis. The first 30 amino acids were omitted from HERC3 and HERC4 and the first 23 amino acids from HERC5 and HERC6. The accession numbers used were as follows: for HERC3, Homo sapiens (human) (NP_055421.1), Gorilla gorilla gorilla (gorilla) (XP_004039158.1), Pan troglodytes (chimpanzee) (XP_517337.3), Nomascus leucogenys (gibbon) (XP_003265945.1), Papio anubis (baboon) (XP_003898995.1), Saimiri boliviensis boliviensis (squirrel monkey) (XP_003924058.1), Callithrix jacchus (marmoset) (XP_002745644.1), Bos taurus (cow) (NP_001077132.1), Ovis aries (sheep) (XP_004009758.1), Felis catus (cat) (XP_003985228.1), Canis lupus familiaris (dog) (XP_535653.3), Ailuropoda melanoleuca (giant panda) (XP_002913643.1), Equus caballus (horse) (XP_001496703.3), *Callorhinchus_milii* (elephant shark) (XM_007902532.1), Danio rerio (zebrafish) (NM_001145624.1), Takifugu rubripes (pufferfish) (XM_011612933.1), Oreochromis niloticus (tilapia) (XM_005456948.2), Latimeria chalumnae (coelacanth) (XM_006005071.2), Xenopus tropicalis (frog) (XM_002938630.3), Anolis carolinensis (lizard) (XM_008111037.2), Geospiza fortis (finch) (XM_005418124.1), Meleagris gallopavo (turkey) (XM_003205471.2), Gallus gallus (chicken) (XM_015276202.1), Monodelphis domestica (opossum) (XM_007495699.2), Mus musculus (mouse) (NM_028705.3), and Dasypus novemcinctus (armadillo) (XM_004480548.2); for HERC4, human (NP_071362.1), gorilla (XP_004049535.1), chimpanzee (XP_001167753.1), gibbon (XP_003258260.1), baboon (XP_003903924.1), squirrel (XP_003928728.1), marmoset (XP_002756356.1), cow (NP_001070362.2), sheep (XP_004021455.1), dog (XP_849808.1), panda (XP_002913788.1), horse (XP_001503636.1), elephant shark (XM_007897346.1), zebrafish (XM_005173035.3), pufferfish (XM_011602912.1), tilapia (XM_005473848.2), coelacanth (XM_005992449.2), frog (NM_001128650.1), lizard (XM_008115175.2), finch (XM_005428501.2), turkey (XM_010714265.1), chicken (XM_015278711.1), Ornithorhynchus anatinus (platypus) (XM_007667149.1), opossum (XM_016421882.1), mouse (NM_030114.2), armadillo (XM_012529817.1), and Petromyzon marinus (lamprey) ENSPMAG00000001626; for HERC5, human (NP_057407.2), chimpanzee (XP_003310459.1), gorilla (XP_004039179.1), marmoset (XP_002745648.1), baboon (XP_003898997.1), squirrel monkey (XP_003924055.1), gibbon (XP_003265940.1), horse (XP_001915115.2), giant panda (XP_002913645.1), sheep (XP_004009762.1), cow (NP_001095465.1), dog (XP_535652.3), cat (XP_003985249.1), coelacanth (XM_014498805.1), lizard (XM_008111035.2), and armadillo (XM_012520464.1); for HERC6, human (NP_060382.3), gorilla (XP_004039178.1), chimpanzee (XP_001160851.1), gibbon (XP_003265938.1), baboon (XP_003899001.1), squirrel (XP_003924053.1), marmoset (XP_002745681.1), cow (NP_001179573.1), sheep (XP_004010096.1), cat (XP_003985250.1), dog (XP_851549.1), horse (XP_001494887.1), pufferfish (XM_011618146.1), tilapia (XM_005474674.1), coelacanth (XM_014498807.1), frog (XM_002938624.2), lizard (XM_008111034.2), opossum (XM_007495704.1), mouse (NM_025992.2), and armadillo (XM_012520459.1).

### Synteny.

Synteny maps were derived using the following reference assemblies: Callorhinchus milii 6.1.3 GCF_000165045.1 (elephant shark), Danio rerio GRCz10 GCF_000002035.5 (zebrafish), Takifugu rubripes FUGU5 GCF_000180615.1 (pufferfish), Oreochromis niloticus Orenil1.1 GCF_000188235.2 (tilapia), Latimeria chalumnae LatCha1 GCF_000225785.1 (coelacanth), Xenopus tropicalis Xtropicalis_v7 GCF_000004195.2 (frog), Anolis carolinensis AnoCar2.0 GCF_0000090745.1 (lizard), Geospiza fortis GeoFor_1.0 GCF_000277835.1 (finch), Meleagris gallopavo Turkey_5.0 GCF_000146605.2 (Turkey), *Gallus_gallus*-5.0 GCF_000002315.4 (chicken), *Ornithorhynchus_anatinus*-5.0.1 GCF_000002275.2 (platypus), Monodelphis domestica MonDom5 GCF_000002295.2 (opossum), Dasypus novemcinctus Dasnovv3.0 GCF_000208655.1 (armadillo), Loxodonta africana Loxafr3.0 GCF_000001905.1 (elephant), Mus musculus GRCm38.p3 GCF_000001635.23 (mouse), Rattus norvegivcus Rnor_6.0 GCF_000001895.5 (rat), Elephantulus edwardii EleEdw1.0 GCF_000299155.1 (shrew), Chrysochloris asiatica ChrAsi1.0 GCF_000296735.1 (cape golden mole), Canis lupus familiaris CanFam3.1 GCF_000002285.3 (dog), and Homo sapiens GRCh38.p2 GCF_000001405.28 (human).

### Positive selection.

Positive-selection analysis was performed as previously described (29). HERC sequences were aligned, and a phylogenetic tree was generated using COBALT (constraint-based alignment tool) (http://www.ncbi.nlm.nih.gov/tools/cobalt/) (84). The following HERC sequences were obtained from GenBank: for HERC3, Homo sapiens (human) (NP_055421.1), Gorilla gorilla gorilla (gorilla) (XP_004039158.1), Pan troglodytes (chimpanzee) (XP_517337.3), Nomascus leucogenys (gibbon) (XP_003265945.1), Papio anubis (baboon) (XP_003898995.1), Saimiri boliviensis boliviensis (squirrel monkey) (XP_003924058.1), Callithrix jacchus (marmoset) (XP_002745644.1), Bos taurus (cow) (NP_001077132.1), Ovis aries (sheep) (XP_004009758.1), Felis catus (cat) (XP_003985228.1), Canis lupus familiaris (dog) (XP_535653.3), Ailuropoda melanoleuca (giant panda) (XP_002913643.1), and Equus caballus (horse) (XP_001496703.3); for HERC4, human (NP_071362.1), gorilla (XP_004049535.1), chimpanzee (XP_001167753.1), gibbon (XP_003258260.1), baboon (XP_003903924.1), squirrel (XP_003928728.1), marmoset (XP_002756356.1), cow (NP_001070362.2), sheep (XP_004021455.1), dog (XP_849808.1), panda (XP_002913788.1), and horse (XP_001503636.1); for HERC5, human (NP_057407.2), chimpanzee (XP_003310459.1), gorilla (XP_004039179.1), marmoset (XP_002745648.1), baboon (XP_003898997.1), squirrel monkey (XP_003924055.1), gibbon (XP_003265940.1), horse (XP_001915115.2), giant panda (XP_002913645.1), sheep (XP_004009762.1), cow (NP_001095465.1), dog (XP_535652.3), and cat (XP_003985249.1); for HERC6, human (NP_060382.3), gorilla (XP_004039178.1), chimpanzee (XP_001160851.1), gibbon (XP_003265938.1), baboon (XP_003899001.1), squirrel (XP_003924053.1), marmoset (XP_002745681.1), cow (NP_001179573.1), sheep (XP_004010096.1), cat (XP_003985250.1), dog (XP_851549.1), and horse (XP_001494887.1). At least 2 independent sequences were available for human, sheep, baboon, marmoset, gibbon, and squirrel monkey. The following sequences were not independently validated: cat, dog, cow, horse, sheep, and giant panda. The identification of site-specific positive selection and purifying selection was generated using the Selecton server (http://selecton.tau.ac.il/index.html). The HERC5 phylogenetic tree was used in the Selecton analysis. Nested pairs of models (M8a and M8; M7 and M8) and a nonnested pair (M8a and MEC) were compared using the likelihood ratio test implemented in the Selecton program.

### Plasmids, transfections, antibodies, and Western blotting. (i) Plasmids.

Plasmids encoding Flag-tagged HERC3, HERC4, HERC5, and HERC6 were created by first PCR amplifying the various HERC coding regions from their respective templates (as described above) using the following primers: for HERC3, forward, 5′ ACG TGA ATT CCA TGT TAT GTT GGG GAT ATT GG 3′, and reverse, 5′ ACG TGG TAC CTC AGG CCA AAC TAA ACC CTT CAT AGT TGT C 3′; for HERC4, forward, 5′ACG TGA ATT CTA TGT TGT GCT GGG GAA ATG C 3′, and reverse, 5′ ACG TTC TAG ATT ATA TTA AAC TGA AGC CTT CAT TGT G 3′; for HERC5, forward, 5′ AAT CGA GAT CTT ATG GAG CGC CGC AGC 3′, and reverse, 5′ TAT GCG GAT CCT CAG CCA AAT CCT CTG 3′; and for HERC6, forward, 5′ AGA TAA GAT CTT ATG TAC TTC TGT TGG GGC 3′, and reverse, 5′ TAG GAG ATA TCT TAT GAC TGT GTG AGC ATG 3′. The amplified products were then cloned into p3xFLAG-CMV10 using the following restriction enzymes: for HERC3, EcoRI and KpnI; for HERC4, EcoRI and XbaI; for HERC5, BglII and BamHI; and for HERC6, BglIII and EcoRV. The resulting plasmids were named pHERC3, pHERC4, pHERC5, and pHERC6.

To generate pH 6:H5RLD, the HERC5 RCC1-like domain was PCR amplified from pHERC5 using the following primers: forward, 5′ GGA TGA CGA TGA CAA GAT GGA GCG CCG CAG CC 3′, and reverse, 5′ TAT GTT CCA GCA AAA ATT ATT AAC TCC TTT TCT GAG GTA TGG CTT TCA AG 3′. The backbone of pHERC6 was PCR amplified using the following primers: forward, 5′ TTT TTG CTG GAA CAT ATG CCA ACT TTG 3′, and reverse, 5′ CTT GTC ATC GTC ATC CTT GTA ATC GAT G 3′. The two amplified fragments were cloned using the fast cloning technique (85).

To generate pH6:H5blade1, blade 1 of HERC5 (amino acids 1 to 100) was PCR amplified from pHERC5 using the following primers: forward, 5′ GGA TGA CGA TGA CAA GAT GGA GCG CCG CAG CCG CCC CAA CAG AAG TAC ATC TTG TCA TCG TCA TCC TTG TAA TCG ATG 3′, and reverse, 5′ GCT CCT TCC CGC AGC TCA CGT GGA TCT TCA TGT TCT TGC CCA GC 3′. The backbone of pHERC6 was PCR amplified using the following primers: forward, 5′ GCT GGG CAA GAA CAT GAA GAT CCA CAG CTG CGG GAA GGA GCA C 3′, and reverse, 5′ GGC TGC GGC GCT CCA TCT TGT CAT CGT CAT CCT TGT AAT CGA TG 3′. The two amplified fragments were cloned using the Gibson cloning technique per the manufacturer's instructions (New England Biolabs).

To generate pH6:R10A, site-directed mutagenesis was performed on pHERC6 using the following primers: forward, 5′ TTC TGT TGG GGC GCC GAC TCC GCG GAG CTG CAG CGC CGG AGG 3′, and reverse, 5′ CCT CCG GCG CTG CAG CTC CGC GGA GTC GGC GCC CCA ACA GAA 3′. pH6:E67A was generated similarly using the following primers: forward, 5′ GCA GCG CGG GGA GCT GCC AGC ACC AAT TCA GGC ATT GGA AAC C 3′, and reverse, 5′ GGT TTC CAA TGC CTG AAT TGG TGC TGG CAG CTC CCC GCG CTG C 3′. pH6:R10A/E67A was generated similarly, except pH6:R10A was used as the template with the following primers: forward, 5′ GCA GCG CGG GGA GCT GCC AGC ACC AAT TCA GGC ATT GGA AAC C 3′, and reverse, 5′ GGT TTC CAA TGC CTG AAT TGG TGC TGG CAG CTC CCC GCG CTG C 3′. pR10G was generated similarly, using the following primers: forward, 5′ CGC CGA CTC CGG GGA GCT GCA 3′, and reverse, 5′ TGC AGC TCC CCG GAG TCG GCG 3′. pHERC3-ΔRLD was generated similarly, using the following primers: forward, 5′ GAC GAT GAC AAG ATG AGC TCA CCA CCA GAT GTT GAA G 3′, and reverse, 5′ CAT CTG GTG GTG AGC TCA TCT TGT CAT CGT CAT CCT TGT AAT CG 3′. pHERC4-ΔRLD was generated similarly, using the following primers: forward, 5′ ACG ATG ACA AGA TGA ATT GGT ACC CCT ATA ATG GGC AGT G 3′, and reverse, 5′ TAG GGG TAC CAA TTC ATC TTG TCA TCG TCA TCC TTG TAA TCG 3′. pHERC5-ΔRLD was generated previously (86). pHERC6-ΔRLD was generated similarly, using the following primers: forward, 5′ CGA TGA CAA GAT GAT TTT TGC TGG AAC ATA TGC CAA C 3′, and reverse, 5′ GTT CCA GCA AAA ATC ATC TTG TCA TCG TCA TCC TTG T 3′. pHERC3-C1018A was generated similarly, using the following primers: forward, 5′ CGG TGG CCC ACA CTG CTT ACA ACC TTC TTG 3′, and reverse, 5′ GAG GTC AAG AAG GTT GTA CGC AGT GTG GGC C 3′. pHERC4-C1025A was generated similarly, using the following primers: forward, 5′ CCC AGT TTC CCA TAC TGC TTT TAA TCT TCT G 3′, and reverse, 5′ GAA GAT CCA GAA GAT TAA AAG CAG TAT GGG AAA C 3′. pHERC5-C994A was generated previously (86). pHERC6-C985A was generated similarly, using the following primers: forward, 5′ CCA ACA TCA ATA ACT GCT CAT AAT ATT CTC TCC C 3′, and reverse, 5′ GGG AGG GAG AGA ATA TTA TGA GCA GTT ATT GAT G 3′.

The promoterless empty-vector plasmid pGL3, p3xFLAG-CMV10, and peGFP were obtained from Promega, Sigma, and Clontech, respectively. pLKO.1/scrambled shRNA and pLKO.1/HERC5 shRNA were previously described (29, 86). The following pLKO.1 shRNA constructs were obtained from Dharmacon: HERC3-#1 (TRCN0000000291), HERC3-#3 (TRCN0000000293), HERC4-#4 (TRCN0000034302), HERC6-#2 (TRCN0000160017), and HERC6-#3 (TRCN0000160044). The coding regions of *HERC3*, *HERC4*, *HERC5*, and *HERC6* were obtained from the following sources: *HERC3* (NM_014606.2), *HERC6* (NM_017912.3) (87), and *HERC5* (NP_057407.2) (33). *HERC4* (NM_015601.3) was isolated from HeLa cells by first reverse transcribing total RNA and then PCR amplification of cDNA using the following primers: forward, 5′ACG TGA ATT CTA TGT TGT GCT GGG GAA ATG C 3′, and reverse, 5′ ACG TTC TAG ATT ATA TTA AAC TGA AGC CTT CAT TGT G 3′. All the constructs were verified by sequencing. Transfections were performed using Lipofectamine 2000 (Invitrogen) according to the manufacturer's instructions unless otherwise indicated. Cotransfections of HERC plasmids with pR9 were performed at a ratio of 10:1 unless otherwise noted. Standard Western blot analyses were performed as previously described (29). Densitometric analysis was performed using ImageJ 1.43u software 64-bit version (NIH).

For the construction of pSIVmac239 (pREC_nfl_SIV239), the SIVmac239 Spx vector was obtained from the NIH AIDS Reagent Program. We previously constructed pREC_nfl_HIV and pCMV_cplt vectors for Saccharomyces cerevisiae-based cloning of diverse HIV-1 strains (88). We developed a similar method for SIV cloning. To generate pREC_nfl_SIV239, the 5′ half of the HIV genome in the pREC_nfl_HIV vector was first replaced with URA3 and then with the 5′ half of the SIV239 genome through the yeast recombination technique described below. Yeast colonies were selected on C-leu plates supplemented with uracil but lacking leucine for selection of pREC_nfl_HIVΔ5′HIV/URA3 and on C-leu supplemented with 5-fluoro-1,2,3,6-tetra-hydro-2,6-dioxo-4-pyrimidine carboxylic acid (5-FOA) for selection of pREC_nfl_5′SIV239/3′HIV. C-leu plates allow growth only when a plasmid containing the leucine gene is transformed into the yeast. The 3′ half of SIV239 was introduced using the same procedure to form the vector pREC_nfl_SIV239; this vector contains nearly the full-length SIV239 genome and lacks the 5′ repeat (R) and unique (U5) regions. Approximately 95% of the FOA-resistant yeast colonies harbored pREC_nfl_SIV239. A crude yeast lysate was then used to transform bacteria and to amplify these ampicillin-resistant DNA plasmids for purification, as described previously (88). For yeast recombination, S. cerevisiae Hanson (MYA-906) (MATα *ade6 can1 his3 leu2 trp1 URA3*) was obtained from the American Type Culture Collection (ATCC). The yeast was grown at 30°C in appropriate medium (yeast extract peptone dextrose [YEPD] or complete [C] minimal medium C-LEU-URA3, C-LEU, or C-LEU/5-FOA), depending on the cloning step. Transformations/recombinations were performed using the lithium acetate (LiAc) method. Briefly, the linearized vector DNA (∼1 μg) and PCR product (∼3 μg) were added to competent cells at a 1:3 ratio, along with 50 μg of single-stranded salmon sperm carrier DNA (BD Biosciences/Clontech, Palo Alto, CA) and sterile polyethylene glycol (50%)-TE (10 mM Tris-Cl, 1 mM EDTA)-LiAc (100 mM). Following agitation for 30 min at 30°C, the yeast was heat shocked at 42°C for 15 min and plated on C-leu agar plates containing the appropriate selection.

### (ii) Antibodies.

The following reagents were obtained through the NIH AIDS Research and Reference Reagent Program, Division of AIDS, NIAID, NIH: HIV-1 p24 monoclonal antibody (183-H12-5C) from Bruce Chesebro and Kathy Wehrly and anti-SIVmac p17 monoclonal antibody (KK59) from Karen Kent and Caroline Powell. Anti-FLAG and anti-hemagglutinin (HA) (clone 3F10) were purchased from Sigma, anti-myc and anti-β-actin were purchased from Rockland, anti-eGFP was purchased from Clontech, and anti-GAPDH (glyceraldehyde-3-phosphate dehydrogenase) (clone 6C5) was purchased from EMD/Millipore. Anti-HERC3 (H00008916-B01P), anti-HERC4 (H00026091-A01), anti-HERC5 (H00051191-A01), and anti-HERC6 (H00055008-A01) were purchased from Abnova.

### Quantitative PCR.

Total RNA was extracted using the PureLink RNA minikit (Ambion, Life Technologies). Three micrograms of RNA was reverse transcribed to cDNA using Moloney murine leukemia virus (MMLV) reverse transcriptase and oligo(dT) primers (Life Technologies). Prior to qPCR, the cDNA samples were diluted 1:5 with water. Each PCR mixture consisted of 10 μl of SYBR green master mix, 2 μl of gene-specific primers (1 μl of 10 μM forward primer and 1 μl of 10 μM reverse primer), 5 μl of diluted cDNA, and water to a total volume of 20 μl. For quantification of incompletely and fully spliced HIV RNAs, qPCR was run on the Rotor-Gene 6000 qPCR machine (Corbett Life Science) under the following cycling conditions: 10 min at 95°C and 40 cycles of 10 s at 95°C, 15 s at 60°C, and 20 s at 72°C. The Rotor-Gene 6000 series software (version 1.7) was used to determine the cycle threshold (*C_T_*) for each PCR. The gene-specific forward and reverse primer sets used were as follows: Gag- (forward, 5′ CAT ATA GTA TGG GCA AGC AGG G 3′; reverse, 5′ CTG TCT GAA GGG ATG GTT GTA G); Rev (forward, 5′ GAG CTC ATC AGA ACA GTC AGA C 3′; reverse, 5′ CGA ATG GAT CTG TCT CTG TCT C 3′). Quantification of endogenous HERC mRNA was run on the QuantStudio5 qPCR machine (Applied Biosystems) under the following cycling conditions: 2 min at 95°C and 40 cycles of 5 s at 95°C, 10 s at 60°C, and 20 s at 72°C. QuantStudio design and analysis desktop software (version 1.4) was used to determine the *C_T_* for each PCR. The primer pairs were as follows: HERC3 (forward, 5′ CAG TGC CCA GGT TAA TAC AAA AG 3′; reverse, 5′ GAA CTC CTT CCC TAA GCC AAG 3′), HERC4 (forward, 5′ TTC ATG TGG AGA AGC TCA TAC G 3′; reverse, 5′ CAT CAG AAT CGA GAC CCC AAG 3′), HERC5 (forward, 5′ ATG AGC TAA GAC CCT GTT TGG 3′; reverse, 5′ CCC AAA TCA GAA ACA TAG GCA AG 3′), HERC6 (forward, 5′ GCG TCA ATT AAG TCA AGC TGA AGC 3′; reverse, 5′ GAA ACC ACA TGC AGG AAC CC 3′), GAPDH (forward, 5′ CAT GTT CGT CAT GGG TGT GAA CCA 3′; reverse, 5′ AGT GAT GGC ATG GAC TGT GGT CAT 3′), and eGFP (forward, 5′ GACAACCACTACCTGAGCAC 3′; reverse, 5′ CAGGACCATGTGATCGCG3′). To ensure no carryover of DNA into each total purified RNA sample, 3 μg of the purified RNA was used directly as the template without reverse transcription for qPCR, using the primer sets described above.

### Statistical analyses.

GraphPad Prism v6 was used for all statistical analyses mentioned in the text. The *P* values and statistical tests used are mentioned where appropriate. *P* values of less than 0.05 were deemed significant.

## Supplementary Material

Supplemental material
