# Supplementary material for: Evolution-Guided Structural and Functional Analyses of the HERC Family Reveal an Ancient Marine Origin and Determinants of Antiviral Activity
Source: J Virol. 2018 Jun 13;92(13):e00528-18. doi: 10.1128/JVI.00528-18 (PMC6002735; doi:10.1128/JVI.00528-18)
Supplement: Supplemental material [file supp_92_13_e00528-18__index.html]

Evolution-Guided Structural and Functional Analyses of the HERC Family Reveal an Ancient Marine Origin and Determinants of Antiviral Activity — Supplemental material 

# Evolution-Guided Structural and Functional Analyses of the HERC Family Reveal an Ancient Marine Origin and Determinants of Antiviral Activity

## Supplemental material

- Supplemental file 1 -

  Table S1 (Parent structure scaffolds used to generate the different structures.)

  PDF, 248K
- Supplemental file 2 -

  Fig. S1 (HERC amino acid identity matrix.)

  XLSX, 57K
- Supplemental file 3 -

  Table S2 (Selection Bayesian results for HERC3 to HERC6.)

  XLSX, 280K
