# Supplementary material for: Evolution-Guided Structural and Functional Analyses of the HERC Family Reveal an Ancient Marine Origin and Determinants of Antiviral Activity
Source: J Virol. 2018 Jun 13;92(13):e00528-18. doi: 10.1128/JVI.00528-18 (PMC6002735; doi:10.1128/JVI.00528-18)
Supplement: Supplemental material [file JVI.00528-18_zjv013183637s1.pdf]

Table S1: Parent structure scaffolds used to generate the different structures.

**H3**	HECT domain	RCC1-like domain	**H5**	HECT domain	RCC1-like domain
Human	1c4z_A	4d9s_A	Panda	1c4z_A	4d9s_A
Horse	1c4z_A	4d9s_A	Squirrel	1c4z_A	4d9s_A
Cat	1c4z_A	4d9s_A	Baboob	1c4z_A	4o2w_A
Chimp	1c4z_A	4dnv_C	Chimp	1c4z_A	4dnv_C
Cow	1c4z_A	4d9s_A	Gibbon	1c4z_A	4d9s_A
Dog	1c4z_A	4d9s_A	Marmoset	1c4z_A	4d9s_A
Gibbon	1c4z_A	4d9s_A	Cow	1c4z_A	4d9s_A
Squirrel	1c4z_A	4d9s_A	Sheep	1c4z_A	4o2w_A
Sheep	1c4z_A	4d9s_A	Human	1c4z_A	4dnu_A
Panda	1c4z_A	4d9s_A	Gorilla	1c4z_A	4o2w_B
Marmoset	1c4z_A	4d9s_A	Horse	1c4z_A	4d9s_A
Gorilla	1c4z_A	4d9s_A	Dog	1c4z_A	4d9s_A
Baboon	1c4z_A	4d9s_A	Lizard	1c4z_A	4d9s_B
Lizard	1c4z_A	4d9s_A	Coelacanth	1c4z_A	4o2w_B
Coelacanth	1c4z_A	4d9s_A	Cat	1c4z_A	4o2w_B

**H4**	HECT domain	RCC1-like domain	**H6**	HECT domain	RCC1-like domain
Baboon	1c4z_A	4d9s_A	Squirrel	1c4z_A	4dnw_A
Squirrel	1c4z_A	4d9s_A	Baboon	1c4z_A	4o2w_B
Sheep	1c4z_A	4dnv_C	Chimp	1c4z_A	4dnv_A
Panda	1c4z_A	4dnv_C	Sheep	1c4z_A	4d9s_A
Marmoset	1c4z_A	4d9s_A	Gibbon	1c4z_A	4dnv_A
Human	1c4z_A	4d9s_A	Cat	1c4z_A	4o2w_B
Horse	1c4z_A	4dnv_C	Horse	1c4z_A	4d9s_A
Gorilla	1c4z_A	4dnw_B	Marmoset	1c4z_A	4jhn_A
Gibbon	1c4z_A	4d9s_A	Cow	1c4z_A	4dnv_A
Dog	1c4z_A	4dnv_C	Human	1c4z_A	4dnv_A
Cow	1c4z_A	4dnv_C	Gorilla	1c4z_A	4o2w_B
Chimp	1c4z_A	4d9s_A	Lizard	1c4z_A	4d9s_A
Lizard	1c4z_A	4d9s_A	Coelacanth	1c4z_A	4d9s_A
Coelacanth	1c4z_A	4dnv_A	Mouse	1c4z_A	4d9s_A
Cat	NA	NA	Dog	1c4z_A	4dnu_A
